# A new convolutional neural network based on combination of circlets and wavelets for macular OCT classification

**DOI:** 10.1038/s41598-023-50164-7

**Published:** 2023-12-19

**Authors:** Roya Arian, Alireza Vard, Rahele Kafieh, Gerlind Plonka, Hossein Rabbani

**Affiliations:** 1 Department of Bioelectrics and Biomedical Engineering, School of Advanced Technologies in Medicine, Isfahan University of Medical Sciences, Isfahan, 81746-73461 Iran; 2 Medical Image and Signal Processing Research Center, Isfahan University of Medical Sciences, Isfahan, 81746-73461 Iran; 3 Department of Engineering, Durham University, South Road, Durham, UK; 4 Institute for Numerical and Applied Mathematics, University of Göttingen, Lotzestr. 16–18, 37083 Göttingen, Germany

**Keywords:** Biomedical engineering, Eye diseases, Computer science

## Abstract

Artificial intelligence (AI) algorithms, encompassing machine learning and deep learning, can assist ophthalmologists in early detection of various ocular abnormalities through the analysis of retinal optical coherence tomography (OCT) images. Despite considerable progress in these algorithms, several limitations persist in medical imaging fields, where a lack of data is a common issue. Accordingly, specific image processing techniques, such as time–frequency transforms, can be employed in conjunction with AI algorithms to enhance diagnostic accuracy. This research investigates the influence of non-data-adaptive time–frequency transforms, specifically X-lets, on the classification of OCT B-scans. For this purpose, each B-scan was transformed using every considered X-let individually, and all the sub-bands were utilized as the input for a designed 2D Convolutional Neural Network (CNN) to extract optimal features, which were subsequently fed to the classifiers. Evaluating per-class accuracy shows that the use of the 2D Discrete Wavelet Transform (2D-DWT) yields superior outcomes for normal cases, whereas the circlet transform outperforms other X-lets for abnormal cases characterized by circles in their retinal structure (due to the accumulation of fluid). As a result, we propose a novel transform named CircWave by concatenating all sub-bands from the 2D-DWT and the circlet transform. The objective is to enhance the per-class accuracy of both normal and abnormal cases simultaneously. Our findings show that classification results based on the CircWave transform outperform those derived from original images or any individual transform. Furthermore, Grad-CAM class activation visualization for B-scans reconstructed from CircWave sub-bands highlights a greater emphasis on circular formations in abnormal cases and straight lines in normal cases, in contrast to the focus on irrelevant regions in original B-scans. To assess the generalizability of our method, we applied it to another dataset obtained from a different imaging system. We achieved promising accuracies of 94.5% and 90% for the first and second datasets, respectively, which are comparable with results from previous studies. The proposed CNN based on CircWave sub-bands (i.e. CircWaveNet) not only produces superior outcomes but also offers more interpretable results with a heightened focus on features crucial for ophthalmologists.

## Introduction

Optical coherence tomography (OCT) is an imaging technique that provides information about the cross-sectional structure of tissues. This non-invasive method has been widely utilized in ophthalmology for investigating retinal diseases and glaucoma, mainly due to the layered structure of the retina^1^. OCT is similar to ultrasound imaging technique; however it uses near-infrared light instead of sound beams^2^.

**Figure 1:**
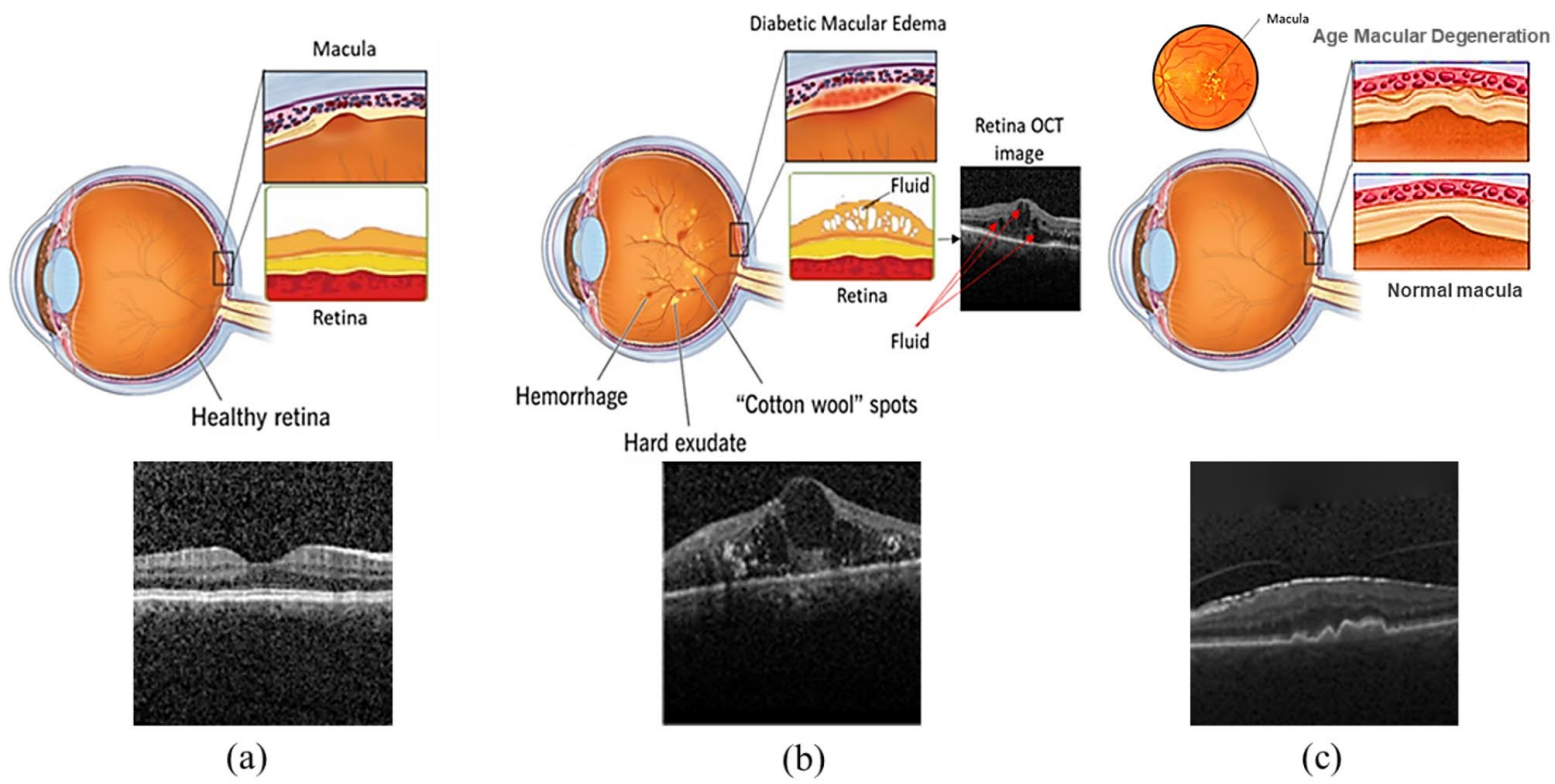
Examples of (**a**) normal, (**b**) DME, and (**c**) AMD eyes along with their corresponding B-scans.

Identifying early symptoms of macular degeneration that affect central vision can prevent vision loss, and OCT images can play an important role in this identification, since they can demonstrate structural changes in the retina. The most common retinal diseases are age-related macular degeneration (AMD) and diabetic macular edema (DME)^3,4^. AMD is a visual disorder caused by retinal abnormalities that reduces the central vision. It occurs when the aging process leads to harm in the macula, which is the section of the eye responsible for overseeing clear, direct vision^5^. DME is another retinal disease associated with diabetic retinopathy and a leading cause of vision loss for people with diabetes. In this abnormality, excess blood glucose damages blood vessels in the retina, causing them to leak. This leakage results in an accumulation of fluid in the macula, causing it to swell^4,6^. Figure 1 shows examples of normal, DME, and AMD eyes along with their corresponding B-scans.

Therefore, OCT can be considered a remarkable biomarker for the quantitation of AMD and DME disorders. Spectral Domain OCT (SD-OCT) and Swept-Source OCT (SS-OCT) represent two newer generations of OCT. Every element in SD-OCT is immobile, resulting in increased mechanical stability and a reduced noise ratio. In contrast to SD-OCT, SS-OCT employs a swept laser light source and photodetector, swiftly producing the interferogram.

**Figure 2:**
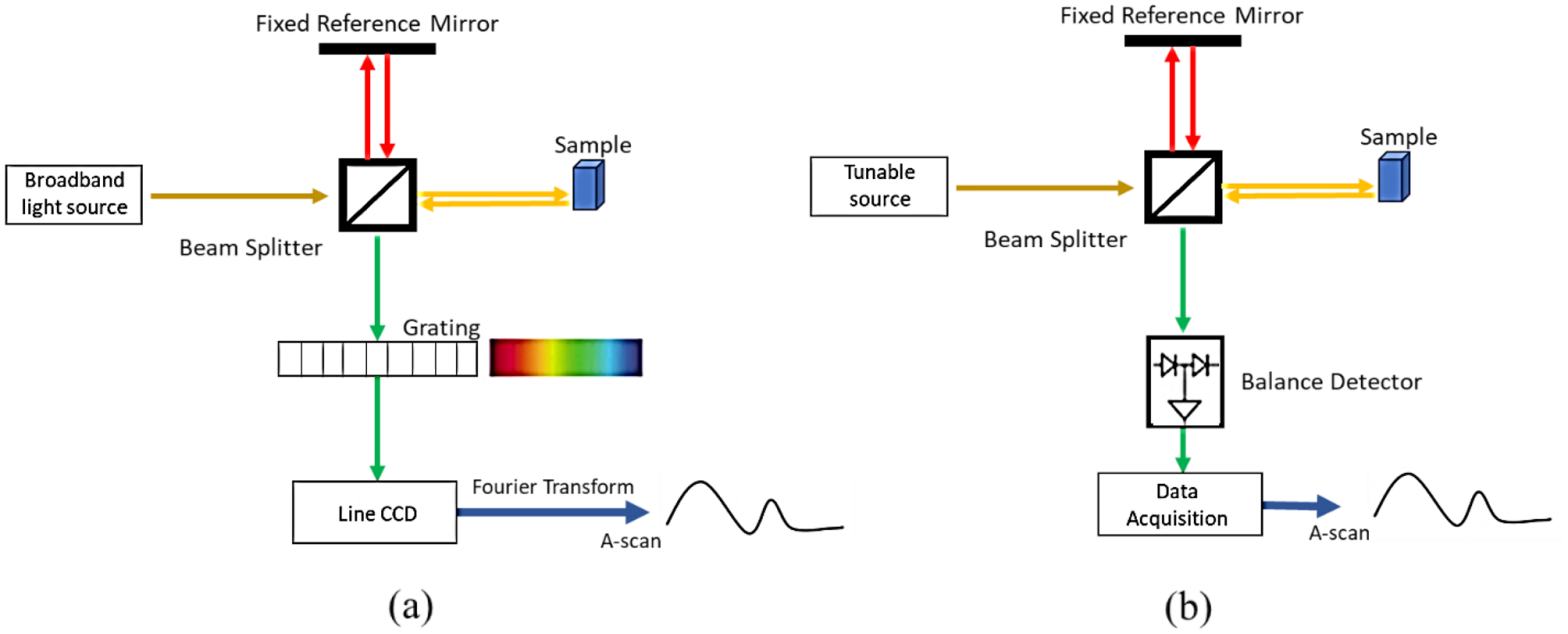
The schematic diagrams of (**a**) SD-OCT, (**b**) SS-OCT.

Figure 2 illustrates the schematic diagrams of SD-OCT, SS-OCT^7^.

Generally, These two systems offer several advantages including a higher rate of acquisition and resolution, reduced light scattering, providing clearer retinal structural information, and improved speed, rendering it better suited for commercial applications^8,9^. Notwithstanding these recent advances in OCT technology, manual analysis remains time-consuming and error-prone due to the similarity of different abnormalities in OCT images. To address these challenges, artificial intelligence (AI) algorithms, including machine learning and deep learning, have been widely employed in image processing for various applications such as classification, segmentation, denoising, and compressive sensing^10–13^. However, several challenges endure in the field of medical imaging while using AI algorithms, often due to the scarcity of data. Thus, image modeling can be utilized alongside AI algorithms for medical image analysis.

Models used in in this field are often rely on appropriate transforms capable of exploiting correlations within the image data, therefore yielding sparse image representations^14^. In other words, crucial information in the data can be efficiently stored using a small number coefficients in the transform domain. Consequently, the classification of this small number of coefficients becomes much easier and faster. Depending on the characteristics of the data and the intended application, the appropriate transform needs to be chosen.

As mentioned in^14^, transform domain approaches can be categorized into data-adaptive and non-data-adaptive models. Regarding classification, non-data-adaptive transforms have the advantage that the information in the transform domain remains comparable. Non-data-adaptive transforms refer to transformations that are obtained without considering the specific nature and structure of the data, and they are computed using a predetermined equation^15^. Among non-data-adaptive models, X-let transforms based on multi-scale time/space-frequency analysis are particularly powerful, as they establish connections between frequency and time information. In X-lets, the original image is decomposed into a set of primary components known as basis functions or dictionary atoms.

These days, non-data-adaptive transformation modeling based on sparse representation has played an important role in various image processing applications. Table 1 provides an overview of recent methods that integrate deep learning with X-let transforms.

**Table 1:** Recent researches on using combination of deep learning and different X-lets for image processing.

References	Year	Application	Dataset	X-let	Combination method	X-let advantages
Darooei et al.^16^	2023	Segmentation	OCT	Contourle, DTCW, Curvelet, Circlet	Application of sparse basis functions as the input for an optimal deep learning architecture	Capturing both local and non-local features of an image simultaneously
Baharlouei et al.^17^	2023	Classification	OCT	WST	Interpreting WST as a convolutional neural network, since it comprises a series of wavelet transform convolutions, along with nonlinear modulus and averaging operators within each layer	Overcoming several shortages in deep learning architectures such as the requirement for using a large training datasets, increasing complexity or processing time; and lack of interpretability
Nur et al.^18^	2023	Classification	CT	Contourlet	Application of Contourlet transform and CNN to extract features individually from segmented images and combining them in one feature vector	Overcoming the limitations of the tensor-product DWT by trying to capture curves rather than points singularities
Tian et al.^19^	2023	Denoising	Natural images (BSD)	Wavelet	Multi-stage image denoising CNN with the wavelet transform via three stages, i.e., a Dynamic Convolutional Block (DCB), two cascaded Wavelet transforms, Enhancement Blocks (WEBs) and a Residual Block (RB)	Improved results compared to some popular denoising methods
Darooei et al.^20^	2022	Segmentation	OCT	DTCW	Application of DTCW sub bands as the input of a U-net	More robust An improved time–frequency analysis of data that has been successfully applied to deep learning segmentation tasks
Wang et al.^21^	2022	Classification	Natural images (Caltech-256)	DTCW & WPT	Application of a CNN model with wavelet domain inputs	Improvement of the network's classification performance significantly
Sarhan et al.^22^	2020	Classification	MRI	Wavelet (Haar)	Extraction of features from images by utilizing the strong energy compactness property exhibited by the Discrete Wavelet Transform and application in a CNN	High reduction of the dimensions of the input image simplifies the work of the CNN classifier
Lakshmanaprabu et al.^23^	2019	Classification	CT images	DWT	Utilizing wavelet features along with histogram and texture features. Then, using the reduced features by Linear Discriminant Analysis (LDA) as the input of Optimal Deep Neural Network (ODNN) which is optimized with Modified Gravitational Search Algorithm (MGSA)	Time saving
Mohsen et al.^24^	2018	Classification	MRI	DWT (Haar)	Application of DWT to extract the features of the segmented tumors and use them as Deep Neural Network (DNN) input	Requires fewer hardware specifications Takes a convenient time for processing large-size images
Khatami et al.^25^	2017	Classification	Radiography images	2D-DWT (Haar)	Application of 2D-DWT to capture the highly discriminative coefficients that represent the complex structure of original data. Then, using the best selected coefficients by Kolmogorov Smirnov (KS) Test as the input of Deep Belief Network (DBN) feature extractor	Time-consuming reduction Increasing accuracy
Rezaeilouyeh et al.^26^	2016	Classification	histopathology images of breast & prostate tissues	shearlet	Extraction of the magnitude and phase of shearlet coefficients and feeding these extra features along with the original images to the CNN	Application of shearlet transform as a general mathematical tool and extracting features without any hand-crafting
Williams et al.^27^	2016	Classification	Natural images (MNIST & CIFAR-10)	Wavelet	Application of preprocessed data in the wavelet domain as the input of CNN	Substantial increase in accuracy compared to the spatial domain processing

*CT* Computed Tomography; *CNN* Convolutional Neural Network; *DWT* Discrete Wavelet Transform; *DTCW* Dual Tree Complex Wavelet; *WST* Wavelet Scattering Transform; *BSD* Berkeley Segmentation Dataset; *WPT* Wavelet Packet Transform; *MRI* Magnetic Resonance Imaging; *MNIST* Modified National Institute of Standards and Technology.

According to Table 1, no prior articles have presented research in the field of image classification using various non-data-adaptive transformations (X-lets). Only a limited number of articles have explored the performance of just one or two transforms in this domain. Therefore, in this study, we aim to evaluate the effectiveness of distinct well-known X-lets with the specific goal of classifying OCT images, intending to establish robust basis functions in this area. The use of a higher number of sub-bands of X-lets results in decreased speed but improved outcomes; hence, a tradeoff is necessary.

Given the focus of the research on OCT images, it is preferable to employ transforms that have demonstrated effective performance in this context. According to Khodabandeh et al.^28^ and taking into account the geometric structure of OCT images, primarily characterized by lines at zero and ± 45 angles, it appears that 2D discrete wavelet transform (2D-DWT), dual tree complex wavelet (DTCW) transform, and contourlet transform exhibit the capability to adequately decompose these images, offering desirable features. In their research, Circlet transform and Ellipselet transform also demonstrated commendable performance in certain evaluation parameters. While Khodabandeh et al.^28^ offered a relatively comprehensive analysis of the effectiveness of these transforms in noise reduction application, it's essential to acknowledge that the application of X-let sub-bands in image classification task is distinctly different. In classification, the objective is to utilize all acquired basis functions simultaneously, requiring the management of different sizes to be incorporated together as input for the feature extraction model. Khodabandeh et al.^28^ did not encounter this challenge as all denoising processes, such as thresholding on the coefficients of X-lets, were conducted separately on each basis, eliminating the necessity to use them concurrently in model training. Furthermore, in that study, basis functions were not utilized as features or inputs for the models. Consequently, there was no necessity to integrate X-lets with deep learning models. On the other hand, Darooei et al.^16^ focused on determining the most effective X-let basis for enhancing OCT cyst semantic segmentation within deep learning methods. They incorporated all basis functions from each X-let transform simultaneously into the Trans-Unet model. However, it is important to recognize that segmentation and classification applications vary, and the optimal X-let transform may differ significantly, given the distinct features and evaluation metrics involved in these two applications. Additionally, in segmentation tasks, there is no requirement to split the test and train data based on subjects, but in classification, any leakage between test and train data subjects can influence the results. Furthermore, neither of the two mentioned studies investigated how altering the number of stages in each transform affects image processing.

It is worth noting that the studies listed in Table 1, which concentrated on classification applications, typically employed the features extracted from X-let transforms coefficients for classification rather than utilizing the sub-bands directly. These aspects distinguish the current study from the aforementioned ones, highlighting the innovations introduced in the current research.

Subsequently, in this study, we will explore the suitability of various mentioned X-lets and their different stages within OCT images for the classification of AMD, DME, and normal B-scans. Moreover, we will directly utilize the obtained sub-bands rather than relying on features extracted from them. This atomic modeling approach applied to OCT images extracts specific nearly optimal basis functions from OCT data, which can be subsequently used for more accurate results in further similar image processing tasks. Additionally, the most effective non-data-adaptive atoms can be utilized as the initial dictionary for a dictionary learning method to closely adapt the basis functions with the data. Given the substantial input dimensions of the classifier model, we will implement a CNN as a dimension reduction model. Furthermore, we anticipate that certain transforms may perform better for classifying all classes except one (e.g., class x), while a different transform might exhibit optimal performance for class x. Therefore, combining these basis functions can provide advantages in achieving comprehensive classification results. We also consider that data splitting should be done subject-wise, where all images belonging to one subject are exclusively assigned to either the test or train dataset.

Therefore, in this present study, the initial step involves transforming all B-scans into various sparse multi-scale X-let transforms. Subsequently, all the resulting sub-bands from every X-let are simultaneously employed as the input for a designed CNN model to extract and identify optimal features. These features are then fed into Multi-Layer Perceptron (MLP) and Support Vector Machine (SVM) classifiers separately for classification purposes.

The rest of this paper is organized as follows: Section “Materials and methods” describes the utilized datasets, X-let transforms, proposed CNN, and classifiers. Section “Experimental results”, and “Discussion” present experimental results and provide discussions, respectively. Our work is concluded in Section “Conclusion”.

## Materials and methods

### Databases

Both datasets used in this study are publicly available, meaning that they are not proprietary or confidential. Ethical considerations, including privacy and confidentiality, were diligently observed to ensure responsible and respectful utilization of the datasets in the research. The data gathering procedures strictly adhered to the principles outlined in the Declaration of Helsinki. In addition, this study was approved by the human ethics board of the Isfahan University of Medical Sciences (approval no. IR.MUI.REC.1400.048).

#### Heidelberg dataset

The first dataset (Dataset-A) used in this study was acquired and collected by Heidelberg SD-OCT imaging systems at Noor Eye Hospital in Tehran^29^. This dataset consists of 50 normal (1535 B-scans), 48 AMD (1590 B-scans), and 50 DME (1054 B-scans) subjects. Details regarding the data are summarized in the supplementary, Table 1.

#### Basel dataset

The second dataset (Dataset-B) utilized in this research was collected in Didavaran eye clinic in Isfahan, Iran, using an SS-OCT imaging system designed and built in Department of Biomedical Engineering at the University of Basel^30^. According to the classification application that is the main goal of our investigation, we chose the “Aligned-Dataset QA” which has been obtained after image contrast enhancement, denoising, and alignment of raw data, respectively^30^.

In this study, 17 cases of DME (2338 B-scans), 15 cases of Non-diabetic (2492 B-scans), and 19 cases of normal (2169 B-sacns) were manually selected from the available subjects (40, 50, and 34, respectively). These cases were chosen because their B-scans are nearly clear and suitable for the classification application.

### Data preprocessing

**Figure 3:**
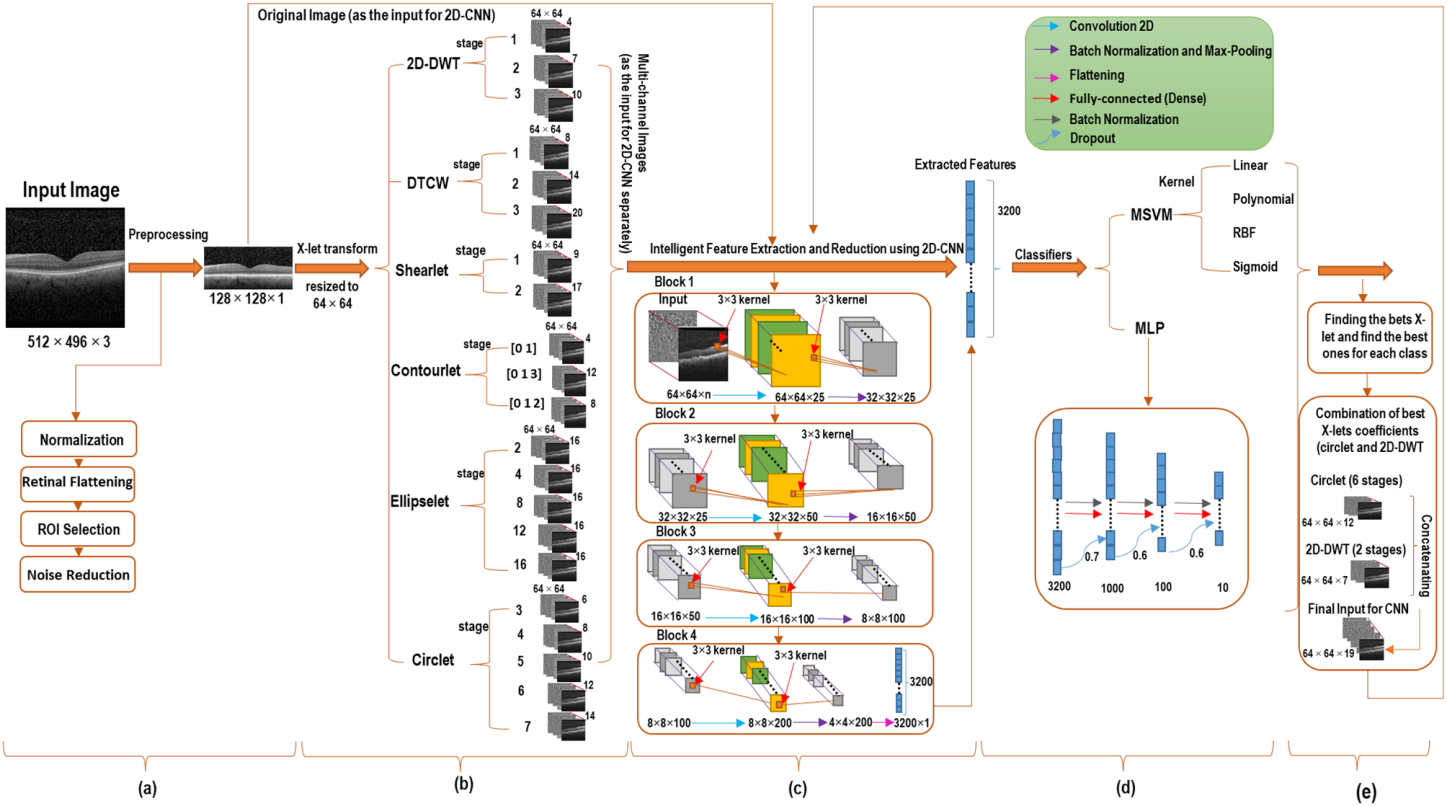
The framework of the proposed classification methods. Where parts (**a**)–(**d**) indicate the steps of preprocessing, transformation, feature extraction, and classification respectively, and part (**e**) represents the proposed transform (combination of circlet and 2D-DWT).

According to Rasti et al.^29^, who presented a suitable preprocessing algorithm for Dataset-A, we applied the following steps in this study the distinction lies in the fact that our investigations are based on a B-scan rather than a volume. Additionally, we incorporated a denoising step into this preprocessing algorithm, as experimental evidence has shown that it produces better results. Figure 3a illustrates the various steps of the preprocessing algorithm.

1. Normalization: Initially, all B-scans were resized to 496 × 512 × 1 pixels to make the field of view of OCT images unique. Subsequently, data normalization was applied by dividing each B-scan by 255, ensuring that all pixel intensities fall within the range [0, 1].
2. Retinal Flattening: Subsequently, a curvature correction algorithm^31^ was used, wherein the hyper-reflective-complex (HRC) is identified as the whole retinal profile, and then localized using the graph-based geometry.
3. Region of Interest (ROI) Selection: Then each B-scan was vertically cropped by selecting 200 pixels above and 200 pixels below the detected HRC. These values were manually chosen to concentrate on the region of the retina containing the primary morphological structures while preserving all retinal information. Following this, the cropped B-scans were resized to 128 × 512 pixels, and the ROI for each one was determined by cropping a centered 128 × 470 pixel sub-image. Finally, each selected ROI was resized to 128 × 128 pixels for further processes.
4. Noise Reduction: Noise Reduction: Finally, denoising of the data was achieved using a non-local means algorithm with a deciding filter strength of 10.

For the “Aligned-Dataset QA” employed as Dataset-B, certain preprocessing steps such as Retinal Flattening and Noise Reduction had already been completed. The size of each B-scan was initially 300 × 300 pixels. Consequently, each B-scan was divided by 255, followed by horizontal cropping that omitted the first 50 pixels (as they contained no remarkable information). Finally, every B-scan was resized to 128 × 128 pixels.

Example B-scans from each class for both datasets, before and after preprocessing, are presented in the supplementary file Fig. 1.

### Splitting training-set and test-set

Any correlation between test and train images can cause bias and impact the results. Therefore, to prevent this undesired leakage, all the images belonging to one subject should be exclusively considered either as test-data or training-data. Ultimately, test data and train data were divided using fivefold nested-cross-validation.

### Classification strategy

#### X-let transforms

The primary purpose of this study is to compare the impact of various geometrical X-let transforms, in two or higher dimensions, on OCT classification. These transforms, furnished by directional time–frequency dictionaries^15^, offer valuable insight into the spatial and frequency characteristics of an image. X-lets are available mathematical tools that provide an intuitive framework for the representation and storage of multi-scale images^27^.

**Figure 4:**
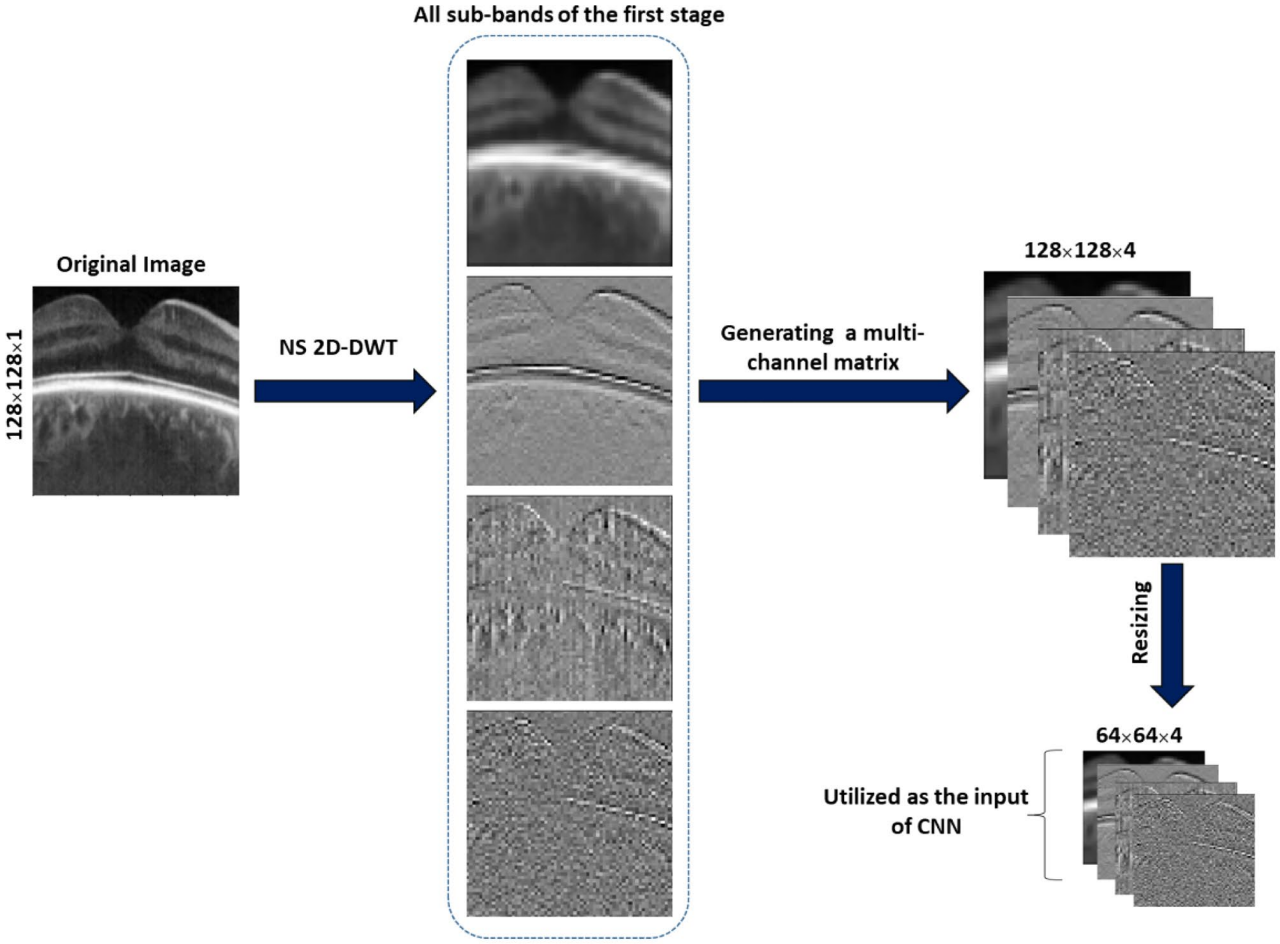
Generating a multi-channel matrix involves concatenating all the sub-bands of the desired transform and preparing it for as input for the CNN.

Therefore, in this study, several geometrical X-let transforms, including 2D discrete wavelet transform (2D-DWT)^14,32,33^ (Note that Haar wavelet was used in the current study), dual tree complex wavelet transform (DTCWT)^20,34^ (Note that just the real parts of this transform are utilized in this research to reduce complexity and redundancy), shearlets^35,36^, contourlets^37^, circlets^38^, and ellipselets^15^ were applied to decompose each B-scan into a linear combination of basis functions or dictionary atoms. The details of this step are illustrated in Fig. 3b. The non-subsampled (NS)^39^ form of the multi-scale X-let transforms was employed to construct a multi-channel matrix for each B-scan using all the sub-bands in parallel. Finally, each multi-channel matrix was resized to (64 × 64 × number of channels) pixels to reduce computational complexity and save time. These steps for one stage of the 2D-DWT is shown in Fig. 4. The details of all utilized X-lets are summarized in supplementary Table 2.

#### Intelligent feature extraction

Using a large number of features for an extensive training-set as the input for NN-models can lead to high computational complexity. Therefore, using a suitable feature reduction algorithm can reduce the training time, enhance accuracy by eliminating redundant data, and consequently reduce over-fitting^40^.

Given the 2D nature of the data, it is essential to employ an algorithm capable of extracting 2D features. While most neural networks and deep learning algorithms transform the 2D input into a vector of neurons, 2D-CNNs, optimized for 2D pattern recognition problems, focus on the inherent 2D nature of images^41^. This specialization makes them particularly well-suited for image classification^27,42^. 2D-CNN inherently possesses the ability to extract features from OCT images in all directions, but using the X-let transformed data as its input can effectively control feature extraction, making the process more intelligent.

Therefore, we designed a 2D-CNN, where the last convolutional layer was flattened and employed as final features, which were then fed into the classifiers.

As illustrated in the Fig. 3b and c, multi-channel images obtained using each X-let transform were used as input for the proposed CNN. The architecture of this CNN, depicted in Fig. 3c, comprises four blocks, each consisting of a 2D-convolution-layer (CL), Batch-Normalization (BN), and Maximum-pooling layers. The filter size for each CL was set to 25, 50, 100, and 200 for blocks 1–4, respectively. Additionally, the “ReLU” activation function (AF), zero padding, and a kernel size of 3 × 3 were utilized in each CL. The optimizer and the loss function were tuned to “Adam” and “categorical-cross-entropy”, respectively. The hyper-parameters in this model were manually adjusted, setting the learning-rate to 10^–3^, the batch-size to 8, and the maximum number of epochs to 100, aiming to achieve the highest possible accuracy during the training phase.

#### Classifiers

In the next step, Multi-Class Support Vector Machine (MSVM) and MLP were applied as classifiers separately. MSVM was chosen because it is known as a simple classifier that allowing for the investigation of the importance and the direct effect of each X-let transform in the results. On the other hand, deep-learning-based architectures have been successfully applied recently in the field of biomedical image processing^10,43–45^. Given the use of a 2D-CNN for feature extraction, an MLP algorithm for classifying OCT B-scans can be used, resulting in a fully deep-learning method.MSVM:MSVM:

- MSVM

SVM is a straightforward classifier that provides insight into the performance of different X-lets, predicting two classes by identifying a hyper-plane that best separates them^46,47^. When the data is perfectly linearly separable, Linear SVM is suitable; otherwise, kernel tricks can aid in classification. Kernel functions, such as a radial-basis-function (RBF), polynomial (poly), or sigmoid, try to transform the lower dimension space (which is not linearly separable) into a higher dimension, making it easier to find a decision boundary.

Although SVM was initially designed for binary classification, it can be extended to a multi-class classifier using various techniques. One such technique is known as the One Versus One (OVO) strategy, which was applied in this study. The OVO strategy divides the dataset into one dataset for each class versus every other class, as shown in supplementary Fig. 2. Ultimately, a voting system, determines the accurate class for each B-scan^48^.

The Grid Search algorithm was used to identify the optimal hyper-parameters for each kernel. This algorithm computes the accuracy for each combination of hyper-parameters in each kernel and selects the values that yield the highest accuracy. The tuned values are summarized in supplementary Table 3.

- MLP

MLP is a nonlinear multi-layer feed-forward neural network that follows the supervised learning technique known as the backpropagation learning algorithm^49^. In this study, the output of the CNN was used as the input layer for the MLP. Three hidden layers (fully connected (FC)-BN) with 1000, 100, and 10 neurons, respectively, were used. To mitigate the risk of overfitting, optimized dropout factors of 70%, 60%, and 60% were applied to the respective hidden layers. “ReLU” AF was employed for the hidden layers, while for the output layer, an FC layer with 3 neurons and “Softmax” AF was utilized. The optimizer, loss function and hyper-parameters were selected similarly to those in the proposed CNN.

Figure 3d indicates the architecture of the MLP classifier.

### Classification evaluation

K-fold cross-validation is a potent method for assessing the performance of machine learning models^50^. A reliable accuracy estimation exhibits relatively small variance across folds^51^. However, a drawback of this method is that the split in each fold is performed entirely randomly. To address this issue, Stratified-K-fold cross-validation can be employed, wherein instead of a random split, the division is done in such a way that the ratio between the target classes in each fold is the same as in the full dataset^52,53^. In the current study, a nested form of Stratified-K-fold was used in order to split test, validate and train data in each fold. We have chosen K = 5, therefore, the experiment was conducted five times, and evaluation parameters were calculated in each fold on the test dataset, and the average values across all folds were then reported as the final results.

The following evaluation parameters including accuracy (ACC), sensitivity (SE), specificity (SP), precision (PR), F_1_-score, and area-under-the-Receiver-Operating-Characteristic (ROC) curve (known as ROAUC) were calculated for each X-let transform.

1 $$accuracy = \frac{TP + TN}{{TP + TN + FP + FN}}$$

2 $$sensitivity = \frac{TP}{{TP + FN}}$$

3 $$specificity = \frac{TN}{{TN + FP}}$$

4 $$precision = \frac{TP}{{TP + FP}}$$

5 $$F_{1} = \frac{{2{ } \times { }TP}}{2 \times TP + FP + FN}$$

6 $${\text{AUC }} = \mathop \smallint \limits_{ - \infty }^{ + \infty } TPR\left( t \right) FPR\left( t \right) dt{ }$$

Here TP represents true positive, FN represents false negative, TN represents true negative, and FP represents false positive. TPR and FPR define the true positive rate and false positive rate respectively.

## Experimental results

In the current study, MATLAB R2020a software was used to extract contourlet, circlet, and ellipselet representations. The Keras and Tensorflow platform backend in python 3.7 software environment, were employed to extract 2D-DWT, DTCW, and shearlet coefficients. Additionally, the classification models were implemented in this environment.

**Figure 5:**
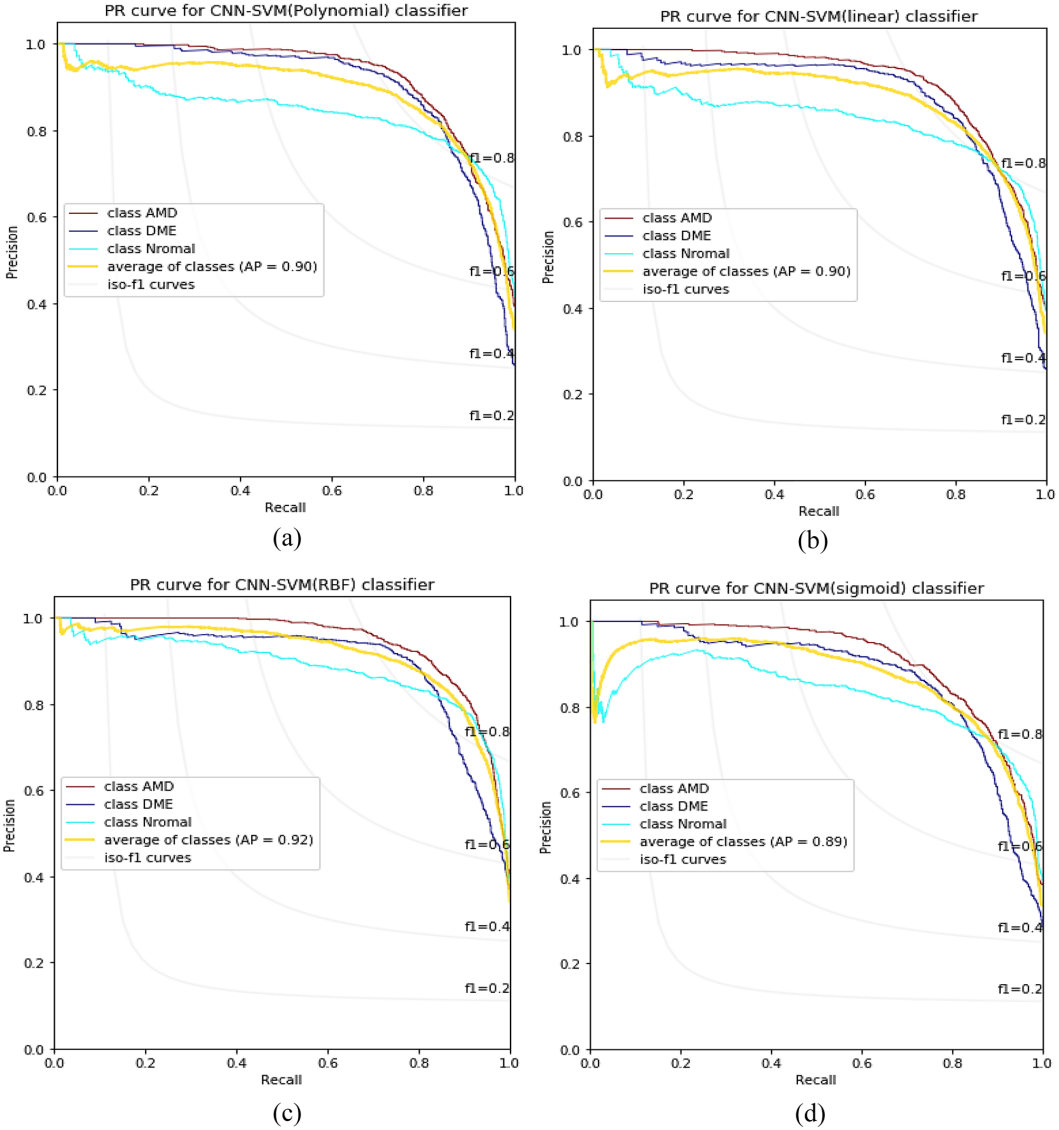
P–R curves of classes for each kernel of MSVM using original Dataset-A. (**a**)–(**d**) represent the polynomial, linear, RBF, and sigmoid kernel respectively.

To determine the optimal kernel for MSVM, precision-recall (P-R) curves for all classes using different kernels were plotted in Fig. 5, with the original Dataset-A considered as the input for MSVM. The P–R curve shows the tradeoff between precision and recall for different thresholds. The average area under these curves for all classes is presented below the curves in each subplot. A high area under P–R curves (PRAUC) signifies both high precision and high recall, corresponding to a low false negative rate and a low false positive rate, respectively. As shown in Fig. 5, the average PRAUC of classes for sigmoid, polynomial, and linear kernels is 0.89, 0.9, and 0.9, respectively. In contrast, it is 0.92 for the RBF kernel, indicating superior performance of this kernel in classifying the dataset.

As the next step, we assessed the performance of different stages of each X-let transform for the classification of Dataset-A using the MLP. Given that RBF was identified as the best kernel for MSVM, evaluation parameters were also reported using the RBF-MSVM and the optimal number of stages for X-lets. This step was repeated using the proposed MLP and RBF classifier, considering the best number of X-let levels for Dataset-B. Table 2 shows these results. As mentioned in Section “X-let transforms”, for the contourlet transform, the decomposition level were presented in a vector.

**Table 2:** Evaluation criteria for different numbers of levels for each X-let transform using the MLP classifier and the combination of the best number of X-let levels and RBF as the best kernel for MSVM classifier. Black bold values show the optimal number of levels for each X-let, while italic values indicate the best values, the superior classifier, and the optimal X-let for OCT classification.

Dataset	Input of classifier	Classifier	Kernel	X-let levels	ACC (%)	SP (%)	SE (%)	PR (%)	F1-score (%)	ROAUC (%)
A	Original	MLP	–	–	88	91	83	85	83	94
		MSVM	RBF	–	89	93	85	86	85	95
	2D-DWT	MLP	–	1	88	91	83	84	84	95
				**2**	89	92	84	85	85	95
				3	88	91	82	84	83	94
		MSVM	RBF	2	90	92	86	87	86	96
	DTCW	MLP	–	1	88	91	83	84	83	94
				**2**	89	92	84	85	84	95
				3	87	90	81	82	81	94
		MSVM	RBF	2	89	91	84	85	84	95
	Shearlet	MLP	–	1	86	89	80	82	79	92
				**2**	88	91	82	83	82	94
		MSVM	RBF	2	88	91	82	83	83	95
	Contourlet	MLP	–	[0 1]	88	91	81	83	82	93
				[0 1 3]	88	91	82	84	82	94
				[0 1 2]	88	91	82	84	82	94
				**[1 2]**	88	92	83	85	83	95
		MSVM	RBF	[1 2]	89	92	84	85	85	95
	Ellipselet	MLP	–	1	89	92	84	86	84	95
				2	87	90	82	83	82	94
				3	90	92	85	86	85	95
				**4**	91	93	86	87	86	96
		MSVM	RBF	4	92	94	87	88	87	97
	*Circlet*	MLP	–	3	90	92	85	86	85	96
				4	88	91	83	84	83	94
				5	91	93	86	86	86	96
				**6**	92	94	87	87	87	97
				7	89	92	84	85	84	94
		*MSVM*	*RBF*	*6*	*93*	*95*	*88*	*89*	*88*	*97*
B	Original	MLP	–	–	83	87	77	79	76	90
		MSVM	RBF	–	84	87	78	80	77	91
	2D-DWT	MLP	–	2	85	89	80	82	80	93
		MSVM	RBF	2	86	90	81	82	79	93
	DTCW	MLP	–	2	86	89	80	82	81	94
		MSVM	RBF	2	87	90	81	82	81	94
	Shearlet	MLP	–	2	81	85	73	73	72	89
		MSVM	RBF	2	83	87	75	80	74	91
	Contourlet	MLP	–	[1 2]	84	88	77	80	75	92
		MSVM	RBF	[1 2]	85	89	79	80	79	93
	Ellipselet	MLP	–	4	88	91	82	83	82	94
		MSVM	RBF	4	88	91	83	84	84	95
	*Circlet*	MLP	–	6	89	91	83	83	83	94
		*MSVM*	*RBF*	*6*	*89*	*92*	*84*	*85*	*84*	*95*

*2D-DWT* 2D Discrete Wavelet Transform; *DTCW* Dual Tree Complex Wavelet; *MLP* Multi-Layer Perceptron; *MSVM* Multi-Class Support Vector Machine; *RBF* Radial-Basis-Function; *ACC* Accuracy; *SP* Specificity; *SE* Sensitivity; *PR* Precision; *ROAUC* Area-under-the-Receiver-Operating-Characteristic curve.

## Discussion

**Figure 6:**
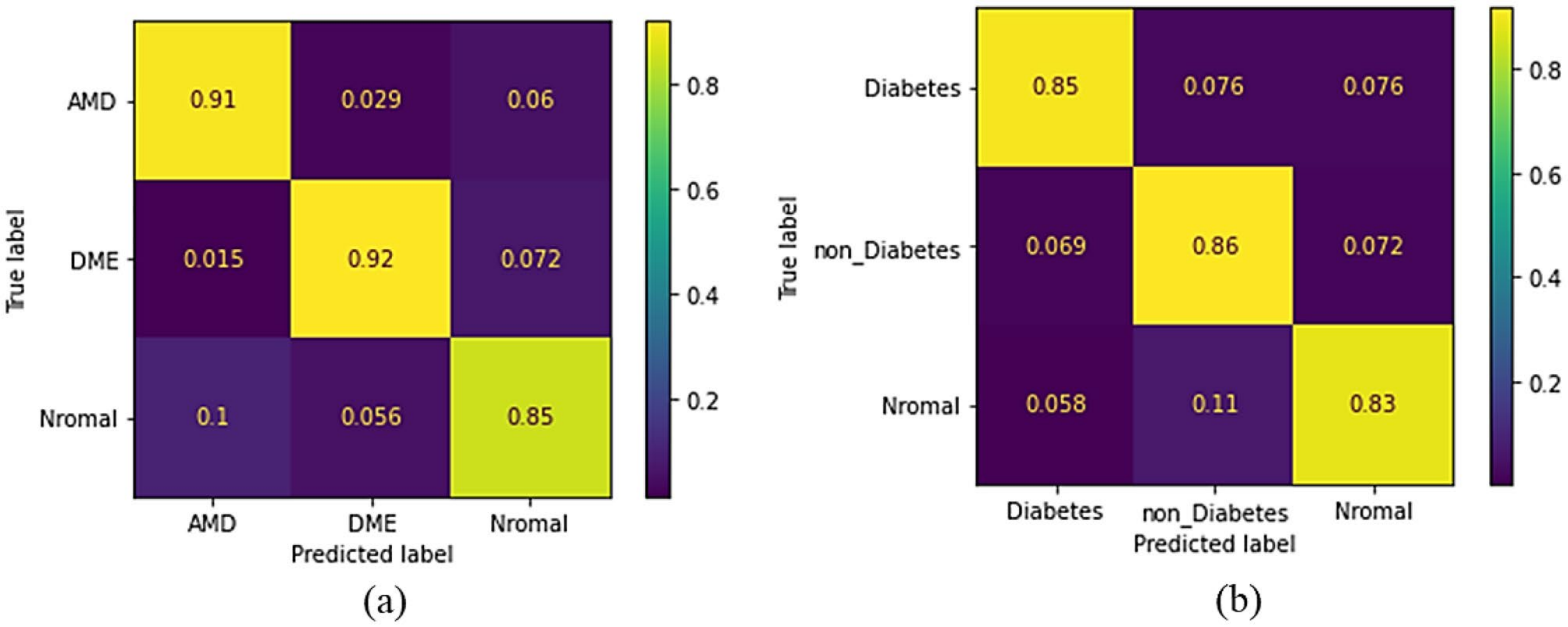
Confusion matrix of the MSVM classifier with RBF kernel for (**a**) Dataset-A and (**b**) Dataset-B. The input to the classifier consists of the circlet basis functions of B-scans.

According to Table 2, it is evident that the circlet transform outperforms other X-let transforms. The optimal confusion matrices, associated with the RBF-MSVM classifier and the circlet transform for both datasets, are shown in Fig. 6.

**Figure 7:**
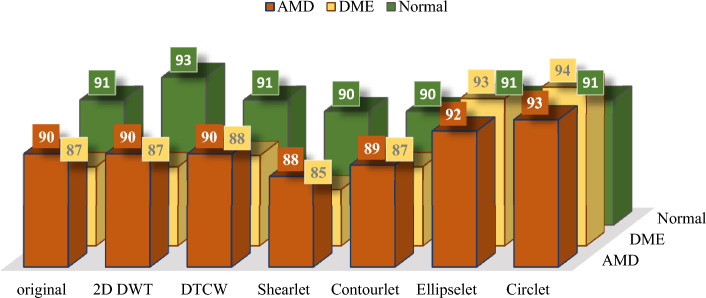
The accuracy of DME, AMD, and normal classes using MSVM with RBF kernel and different X-lets. These values are expressed as a percentage.

**Figure 8:**
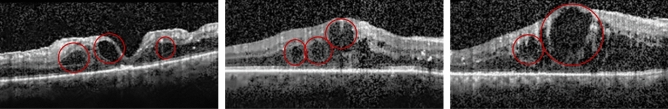
Appearing circles on B-scans of DME subjects. Because of the fluid accumulation.

**Figure 9:**
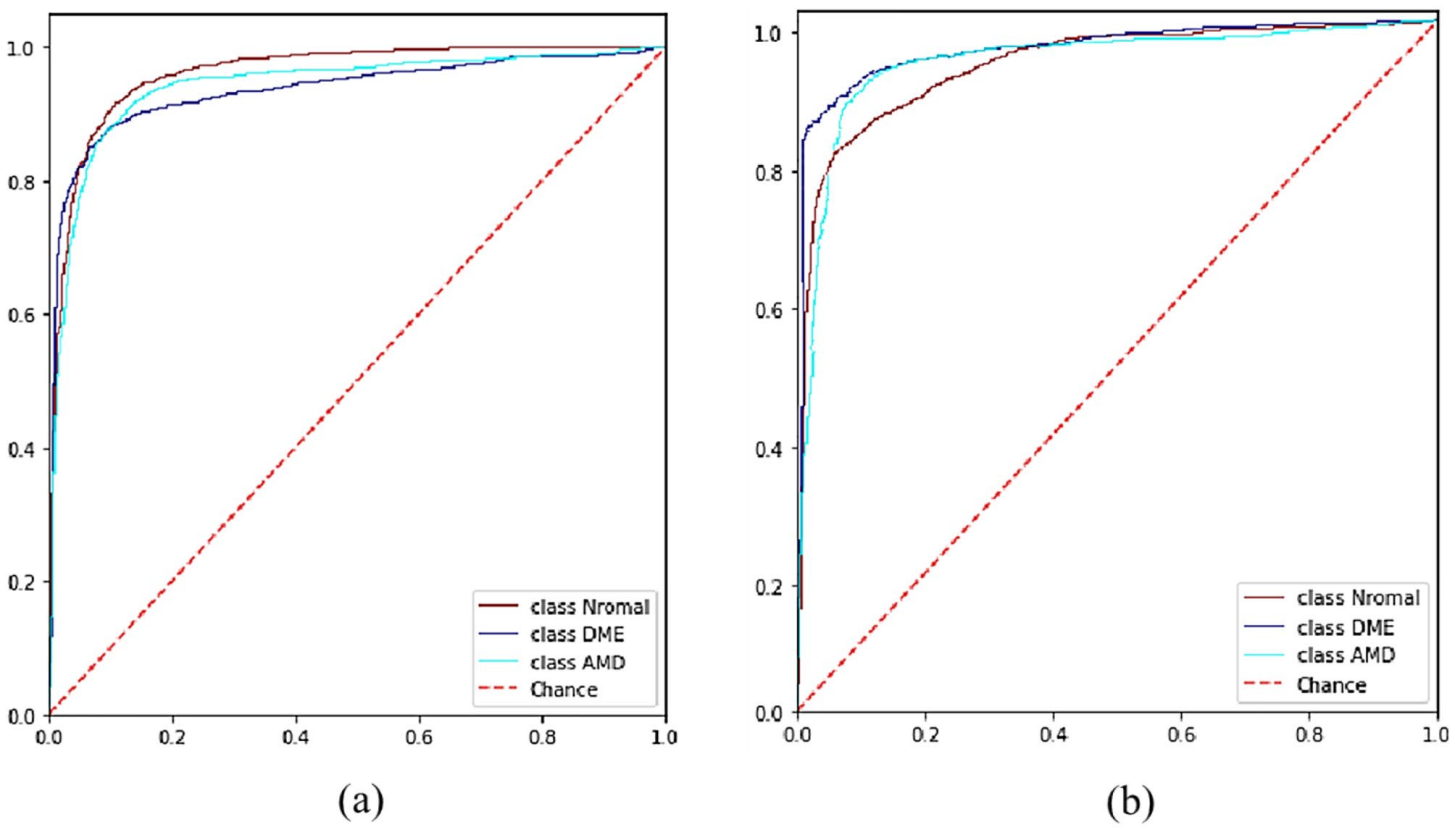
The ROC curves of different classes for (**a**) 2D-DWT, and (**b**) circlet transform. Where the ROC curve of normal, DME, and AMD classes are shown in dark red, purple, and turquoise color respectively.

The accuracy achieved for each X-let transform in the classification of each class individually, is presented in Fig. 7, where the circlet transform demonstrates the best performance for the DME class, while the 2D-DWT provides better results for the normal class. It seems that the appearing circles on DME B-scans can be detected much more effectively using the circlet transform (Fig. 8 shows some of these appearing circles caused by the accumulation of fluid). Moreover, Fig. 9 shows the ROC curves of classes for the circlet transform and the 2D-DWT. It is observed that the ROAUC of the DME class is better using the circlet transform, whereas the normal class has a superior ROAUC using the 2D-DWT because most of the B-scan layers belonging to this class are aligned at 0 degrees.

**Figure 10:**
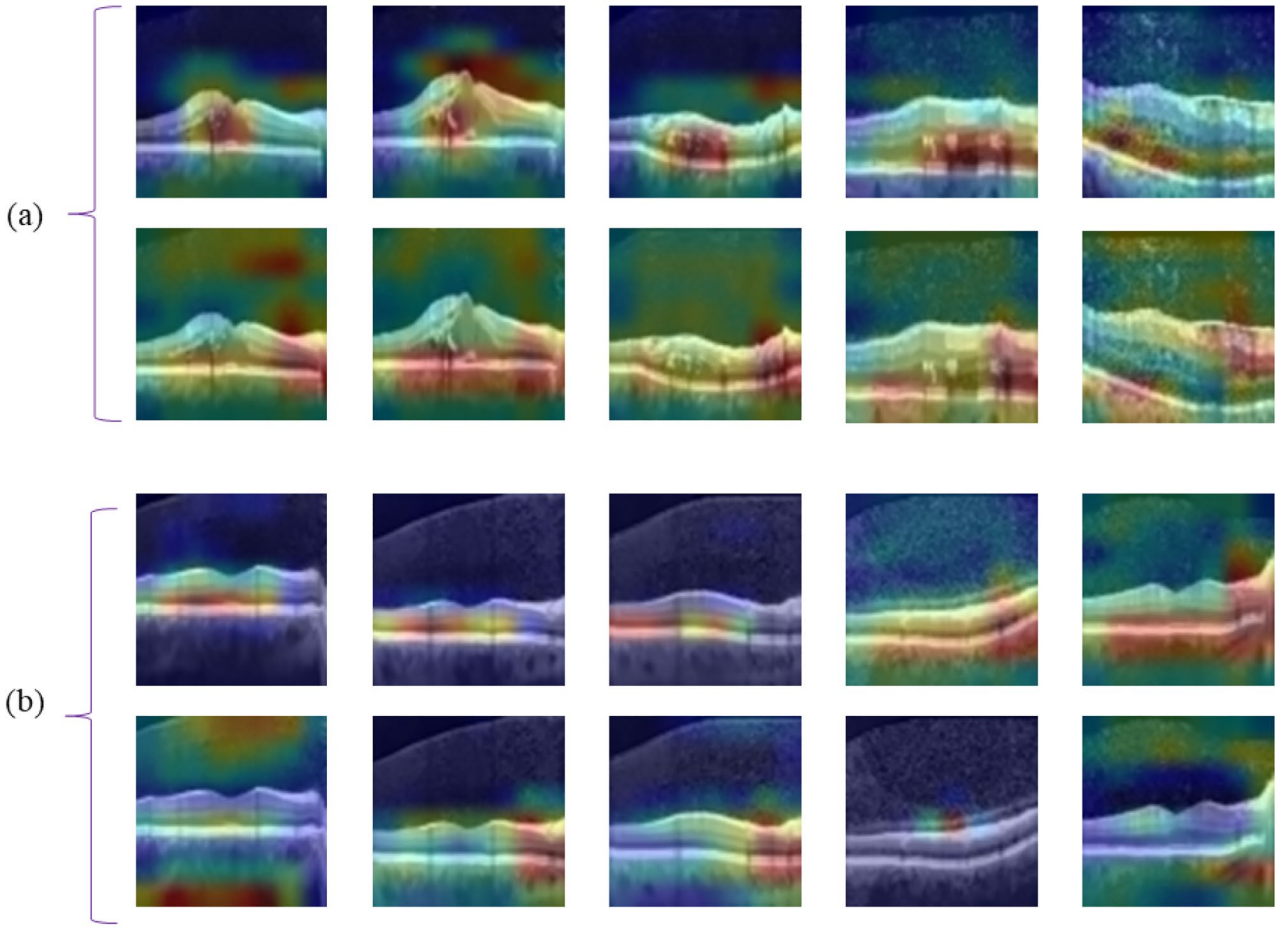
The Grad-Cam of the proposed CNN for several test data of Dataset-A. Where part (**a**) compares the heat maps of reconstructed B-scans using circlet bases (first row) and the associated original B-scans (second row), while part (**b**) compares the heat map of reconstructed B-scans using 2D-DWT bases (first row) and the associated original B-scans (second row). It's important to note that part (**a**) shows DME B-scans and part (**b**) shows normal B-scans.

In order to compare the classification results achieved by employing two transforms (2D_DWT, and circlet) with classification using the original image, the B-scans were reconstructed using half of the sub-bands of each transform individually. The reconstructed B-scans were again utilized as the input of proposed models. Finally, the Grad-CAM class activation visualization was plotted in Fig. 10 for several B-scans using the original B-scans and the reconstructed ones using circlet transform bases (for DME cases) and the 2D-DWT bases (for normal cases), respectively. This heat map can give some perspective of the parts of an image with the most impact on the classification score. For the reconstructed B-scans using the circlet transform these image parts concentrate on appearing circles on DME B-scans, while for the reconstructed B-scans using 2D-DWT, these image parts contain lines in normal B-scans. Notably, these image parts in the original B-scans do not emphasize such characteristics. It's important to note that for all B-scans shown in Fig. 10, the classifier predicted the class correctly using either the original data or the X-let transforms, but the concentrations in the heat maps are entirely different.

Accordingly, it appears that a combination of these two transforms, as shown in Fig. 3e, can offer better performance in the classification of these datasets, as it can simultaneously emphasize the nature of circles in the DME class and straight lines in the normal class. The results of this experiment are reported for both datasets in Table 3. In addition, in this table, the performance of the proposed CNN is compared to VGG16 and VGG19 as two state-of-the-art models. Note that, these values are obtained using MSVM with RBF kernels as the best proposed classifier.

**Table 3:** Performance comparison of the proposed CNN and VGG19 and VGG16 as feature extraction models. These values were obtained using a combination of Circlet and 2D-DWT bases as the input to the models. Italic values represent the best-achieved results for each dataset.

Feature extraction model	Dataset	ACC (%)	SE (%)	SP (%)	PR (%)	F1-score (%)	ROAUC (%)
*Proposed CNN*	A	*94.5*	*96*	*89.5*	*90*	*90*	*98*
	B	*90*	*92*	*84.5*	*86*	*85*	*96*
VGG19	A	93	94.5	88	88	88	96.5
	B	88.5	91	83	84	84.5	95
VGG16	A	91.5	93	87.5	87	87	96
	B	87	90	82	83	82.5	94

*CNN* Convolutional Neural Network; *ACC* Accuracy; *SP* Specificity; *SE* Sensitivity; *PR* Precision; *ROAUC* Area-under-the-Receiver-Operating-Characteristic curve.

According to Table 3, the proposed CNN outperforms VGG19 and VGG16 in feature extraction, despite utilizing significantly fewer trainable parameters. Furthermore, the combination of the circlet transform and 2D-DWT yields better results than using only the circlet transform.

**Figure 11:**
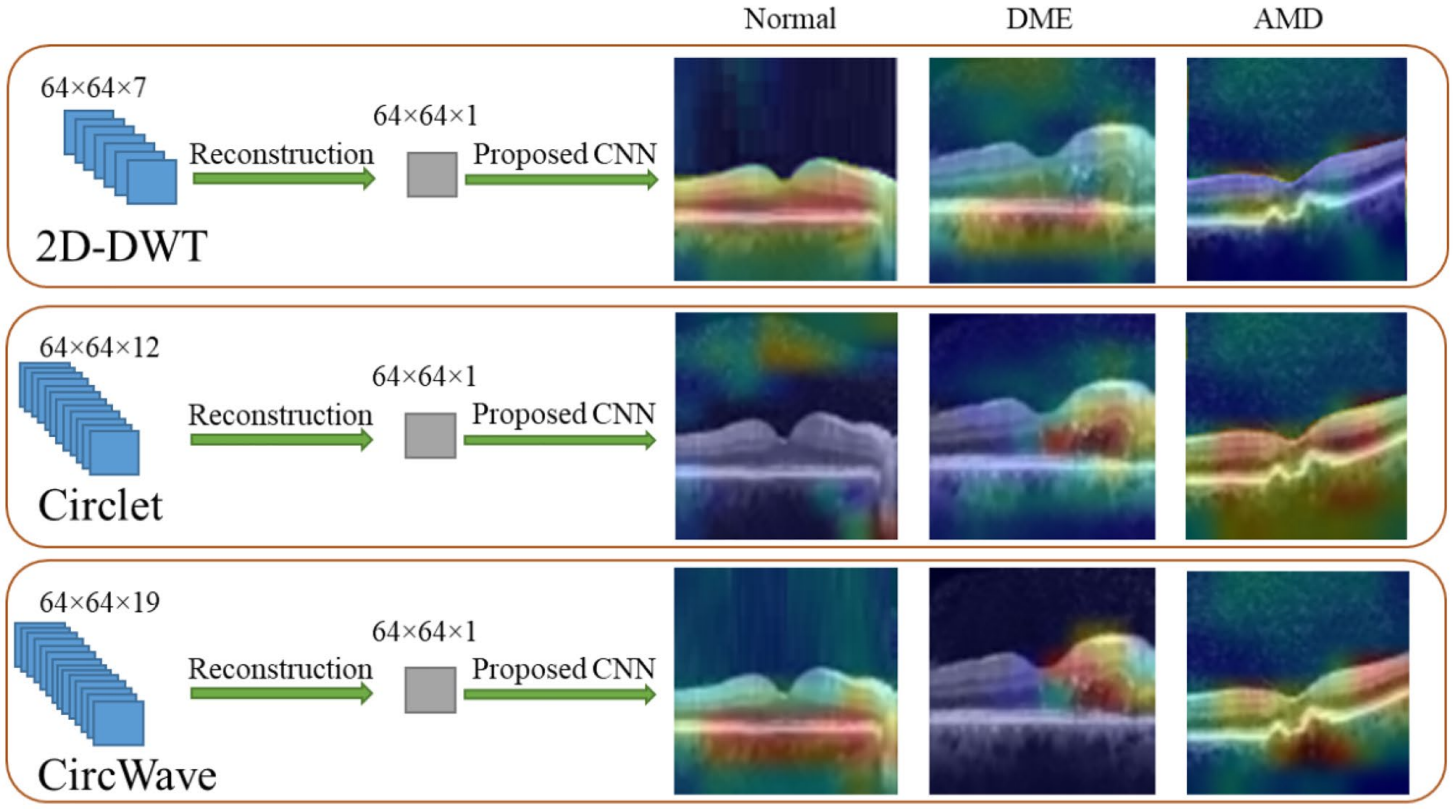
The Grad-Cam of the proposed CNN for three test data of Dataset-A for each class, when reconstructed B-scans using 2D-DWT, Circlet, and CircWave transforms (in first to third rows), respectively are used as the input of the CNN.

We call our proposed transform and method the “CircWave” and the “CircWaveNet”, respectively. To demonstrate the advantages of CircWave compared to Circlet and 2D-DWT, three B-scans from each class were selected and the heat map was plotted in Fig. 11 for each one, where the reconstructed B-scans, utilizing half of the sub-bands of each of the three mentioned transforms separately, served as the input for the CNN. It is clearly evident that for the normal case, 2D-DWT provides a more accurately focused heat map, while for the DME case, Circlet performs better. However, the proposed CircWave transform can concentrate on the correct regions for both normal and DME cases simultaneously, and provide a more suitable heat map for AMD cases as well. Note that these three B-scans were classified correctly using all three mentioned transforms.

**Figure 12:**
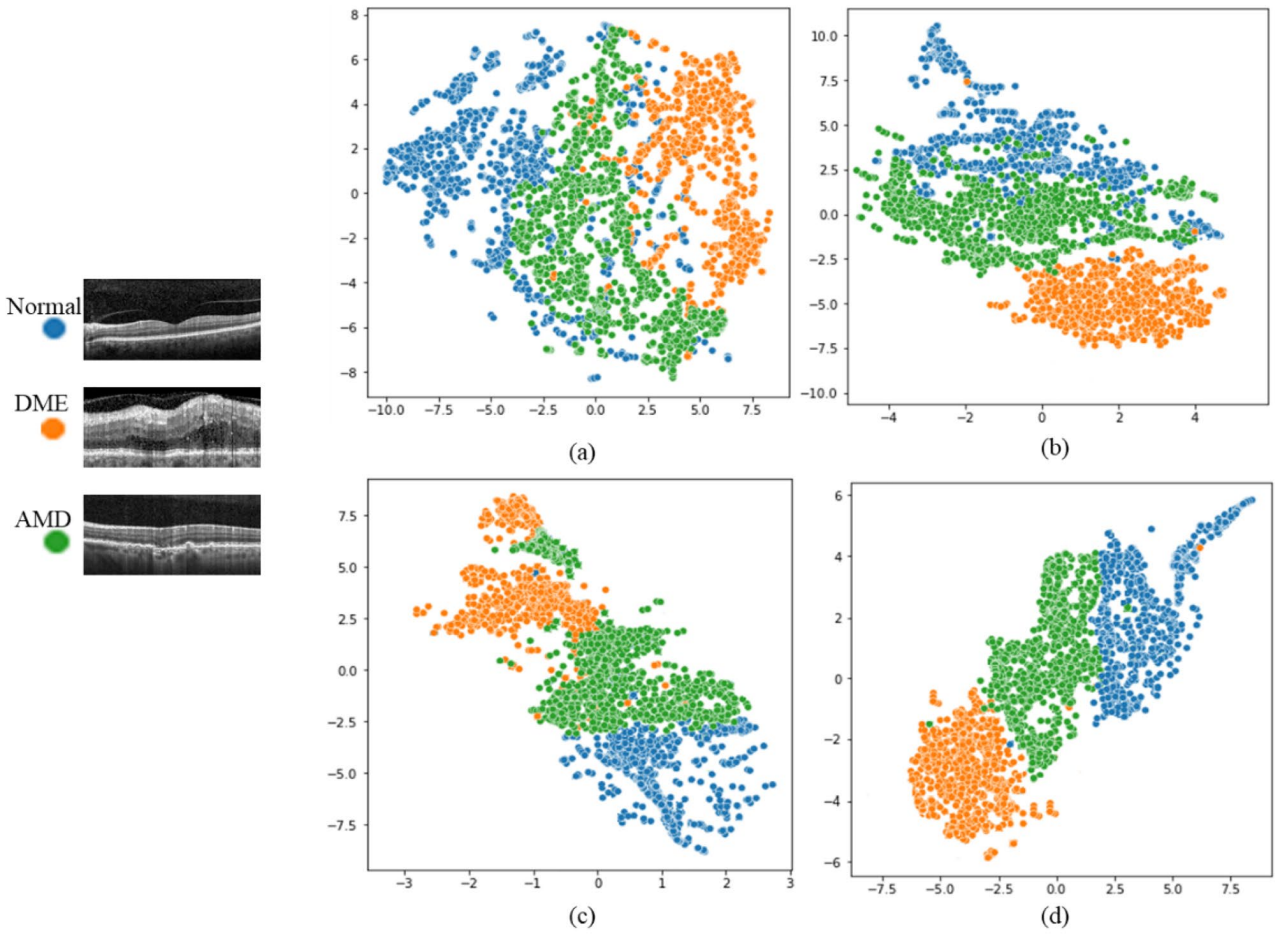
Visualization of the output of the proposed CNN using PCA in conjunction with t-SNE reduction algorithms for (**a**) The original data, (**b**) the Circlet bases, (**c**) the 2D-DWT bases, and (**d**) the CircWave bases.

We also employed principal component analysis (PCA) in conjunction with t-distributed stochastic neighbor embedding (t-SNE) techniques to visualize the high-dimensional outputs of the proposed CNN, when the original data, 2D-DWT transformed data, Circlet transformed data, and CircWave transformed data were used as inputs, respectively. The proposed CNN extracts 3200-dimensional features for each mentioned input. First, the PCA reduction algorithm was employed to reduce the number of dimensions and create a new dataset containing fifty dimensions. Subsequently, they were further reduced to two dimensions using the t-SNE technique. The resulting dataset from each input was then plotted in Fig. 12.

It can be noticed that when CircWave bases are used as the CNN input, samples from all three classes are spaced apart and well grouped together with their respective cases. While the Circlet transform can clearly cluster DME cases in their own class, it struggles to separate normal and AMD cases effectively. Additionally, the 2D-DWT can group normal cases well but faces challenges in properly distinguishing between AMD and DME cases. Notably, the original data is not efficient in providing separable features for this 3-class classification problem.

To the best of our knowledge, this is the first time that Dataset-B has been utilized for classification application. We have demonstrated that the proposed CircWaveNet is successful in classifying this dataset, outperforming results obtained using the original data and other transforms.

On the other hand, there are several research results that use Dataset-A for classification applications. In Table 4, we summarize these results.

**Table 4:** Results from other articles on Dataset-A. Results from the proposed CircWaveNet are shown in bold.

Number	Paper	Dimension	Method	ACC (%)	SE (%)	PR (%)	ROAUC (%)
1	Rasti et al.^29^	3D	Multi-scale Convolutional Mixture of Expert	–	–	99.39	99.8
2	Fang et al.^54^	3D	Lesion-Aware CNN	–	99.36	99.39	99.80
3	Das et al.^55^	3D	B-scan Attentive CNN	93.2	–	–	95
4	Rasti et al.^56^	3D	Wavelet-based Convolutional Mixture of Experts	–	–	–	99.3
5	Wang et al.^57^	3D	Volumetric OCT-Recurrent Neural Network	93.8	94.0	94.4	–
6	Wang et al.^44^	2D	CliqueNet	98.6	–	–	–
7	Das et al.^58^	2D	semi-supervised Generative Adversarial Network	97.43	97.43	–	–
8	Xu et al.^59^	2D	Multi-branch Hybrid Attention Network	99.7	–	1	–
9	Nabijiang et al.^60^	2D	Block Attention Mechanism	99.64	–		–
10	**Our Method**	**2D**	**CircWaveNet**	**94.5**	**96**	**90**	**98**

*CNN* Convolutional Neural Network; *ACC* Accuracy; *SE* Sensitivity; *PR* Precision; *ROAUC* Area-under-the-Receiver-Operating-Characteristic curve.

According to Table 4, some of the results seem superior to those of CircWaveNet, but it is important to note that the first four mentioned articles worked on 3-D volumes. In their method, a specific threshold (like: τ = 15 or τ = 30) is utilized, and if more than τ percentages of B-scans belonging to one subject are predicted as abnormal, the maximum probability of B-scans’ votes (based on AMD or DME likelihood scores) determines the type of patient's retinal disease. The fifth article used volume-level labels for each subject, instead of labeling each B-scan separately. In contrast, in this paper, parameters are determined based on the B-scan, which is inherently more challenging than a subject-based approach.

The other articles (no. 6–9) mentioned in Table 4, along with this paper, focused on 2D B-scan classification. Although these papers achieved better results than our method, it should be noted that, in these articles, the train and test sets are divided according to the ratio of 8:2, regardless of the potential leakage between test and train subjects, which can cause bias and certainly increases the results wrongly. In contrast, in this article, all the images belonging to one subject were considered either as test-data or training-data. This data splitting strategy enhances the reliability of the test results for ophthalmologists.

Additionally, many of the mentioned methods require training a large number of training parameters (more than 100 million), whereas CircWaveNet has approximately 3.5 million total training parameters, significantly reducing the computational complexity.

As stated in the introduction, the optimal selection of an X-let transform can differ across applications, influenced by the utilization of distinct evaluation metrics and various image features that need to be considered. Darooei et al.^16^ determined that, for OCT B-scan segmentation using dice coefficient and Jaccard index, contourlet yields optimal results. Khodabandeh et al.^28^ observed diverse performance in noise reduction, where DTCW transform excels in Structural Similarity Index (SSIM), and 2D-DWT in Edge Preservation (EP) and Texture Preservation (TP). This suggests the effectiveness of different X-lets based on distinct criteria and image characteristics. However, our study demonstrates that CircWave transform surpasses others in both quantitative results and interpretability.

## Conclusion

In this paper, we proposed applying suitable X-let transforms to OCT B-scans rather than the original images as input for the 2D-CNN to achieve improved classification results while significantly reducing computational costs. This is feasible by transferring the data to a transform domain that allows a sparse image representation with a small number of transform coefficients. We have demonstrated that almost all X-let transforms can lead to more accurate classification results than the original B-scans. Among all utilized X-let transforms, the circlet transform performs better for both considered datasets, obtaining 93% ACC, 95% SE, 88% SP, 89% PR, 88% F1-score, and 97% ROAUC in Dataset-A, and 89% ACC, 84% SE, 92% SP, 85% PR, 84% F1-score, and 95% ROAUC in Dataset-B. Concentrating on class-accuracy, we found that the 2D-DWT can perform better for the classification of normal cases because most lines and boundaries in a normal B-scan almost follow a straight pattern with zero degrees, which can be well detected using a simple 2D wavelet transform capable of extracting lines with 0, 90 and ± 45 degrees. However, in the retinal structure of DME cases, some circles appear due to fluid accumulation and an increase in retinal thickness. This characteristic changes the pattern of B-scans of DME cases, which is extracted much better using the circlet transform.

Moreover, this paper demonstrates that these two transformations not only provide a significant increase in evaluation parameters but also focus on the characteristics of each class that are crucial for ophthalmologists. While it is necessary but not sufficient for them to categorize each case, X-lets make this decision more reliable because they concentrate on the true discriminative features of each class. Despite the classifier can predict the class for most B-scans using even original data, the CNN based on the considered X-let transforms precisely focuses on the features that make a difference in classes, a distinction not observed with original data.

As the next step, and to enhance the accuracy of the classification models, the coefficients from the 2D-DWT and circlet transform were concatenated and then fed into the models. This proposed algorithm increased the evaluation parameters by approximately 0.5 to 1.5 percent in both datasets. We named the new transformation "CircWave" and the novel classification model “CircWaveNet”.

This paper demonstrates that, despite the significant advancements in deep learning for image classification, certain limitations persist, especially in domains like medical images where there may be a scarcity of data or labeled data. Therefore, the utilization of image processing techniques, such as the application of time–frequency transforms, along with deep learning methods, can contribute to enhancing the accuracy and reliability of such applications, as they enable the analysis and interpretation of data, facilitating informed decision-making based on accurate information.

In future research, CircWave transform atoms can serve as the initial dictionary for a dictionary learning model, facilitating the adaptation of these atoms to the data. Conventional dictionary learning models are typically initialized with a random start dictionary, constituting an unsupervised initialization. When the goal is classification, there is supposed to be an advantage in applying a supervised initialization approach. Specifically, utilizing an initial dictionary that focuses on the distinct characteristics of various classes may potentially result in superior performance. This data-adaptive technique aims to provide more accurate results compared to X-let transforms, as it generates atoms based on the given data observations, making them better suited to the data. The adapted atoms can be subsequently employed in future image processing applications, ensuring faster and more accurate outcomes.

### Supplementary Information

Supplementary Information.

## Data Availability

Dataset-A can be accessed via: https://misp.mui.ac.ir/en/dataset-oct-classification-50-normal-48-amd-50-dme-0, while Dataset-B is accessible at: https://misp.mui.ac.ir/en/oct-basel-data-0. Code and models are available at: https://github.com/royaarian101/CircWaveNet.
