# Supplementary material for: A new convolutional neural network based on combination of circlets and wavelets for macular OCT classification
Source: Sci Rep. 2023 Dec 19;13:22582. doi: 10.1038/s41598-023-50164-7 (PMC10730902; doi:10.1038/s41598-023-50164-7)
Supplement: Supplementary file 1 — Supplementary Information. [file 41598_2023_50164_MOESM1_ESM.docx]

Supplementary for manuscript titled:

**CircWaveNet: A New Conventional Neural Network Based on Combination of Circlets and Wavelets for Macular OCT Classification**

Roya Arian^1,2^ , Alireza Vard^1^, Rahele Kafieh^3^ , Gerlind Plonka^4^, Hossein Rabbani ^2,1,^*

^1^ School of Advanced Technologies in Medicine, Isfahan University of Medical Sciences, Isfahan, 81746-73461, Iran

^2^ Medical Image and Signal Processing Research Center, Isfahan University of Medical Sciences, Isfahan, 81746-73461, Iran

^3^ Department of Engineering, Durham University, South Road, Durham, UK

^4^ Institute for Numerical and Applied Mathematics, University of Göttingen, Lotzestr. 16–18, D-37083 Göttingen, Germany

* Correspondence: [Rabbani.h@gmail.com](mailto:Rabbani.h@gmail.com)

This supplementary provides detailed information about the data, the proposed method, and the experimental results.

| **Characteristics** | **Value** | **Unit** |
| --- | --- | --- |
| Axial resolution | 3.5 | μm |
| Scan-dimension | 8.9×7.4 | mm^2^ |
| Number of A-scans | 512 to 768 | Scans |
| Number of B-scans per volume (for different patients) | 19, 25, 31, and 61 | Scans |

Table-1. Heidelberg dataset information


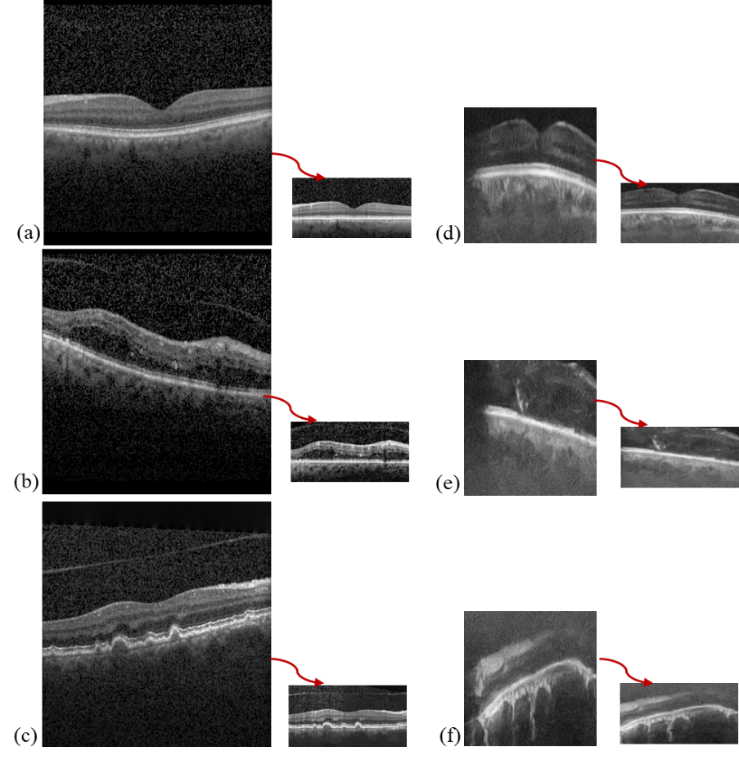


Figure-1. Example B-scans of (a) Normal, (b) DME, and (c) AMD cases in Dataset-A, and (d) Normal, (e) Diabetes, and (f) non-Diabetes in Dataset-B, before and after preprocessing. Red arrows connect the non-preprocessed B-scans to the related preprocessed ones.

Table-2. Properties of Different X-lets

| X-let | Properties | |
| --- | --- | --- |
| 2D-DWT  (NS) | Note | First applying a one level Haar wavelet to each row and secondly to each column |
|  | Filter Bank  (1 stage) | 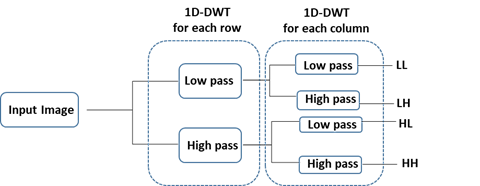 |
| DTCW  (NS) | Note | 1. Using two real DWTs in parallel to create real and imaginary parts of the transform. 2. Producing six sub-bands in six directions (±15, ±45, and ±75) |
|  | Filter Bank  (1 stage) | 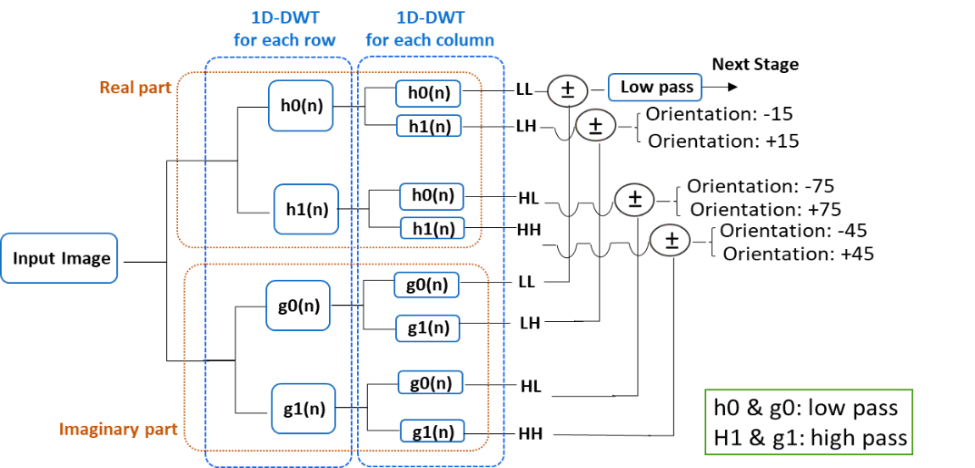 |
| Contourlet  (NS) | Note | 1. Using multi-directional decomposition and multi-scale decomposition for images. 2. Divided into two shift-invariant parts: a Non Subsampled Pyramid structure (that ensures the multiscale property) and a Non Subsampled Directional Filter Bank structure (that gives directionality). |
|  | Filter Bank | 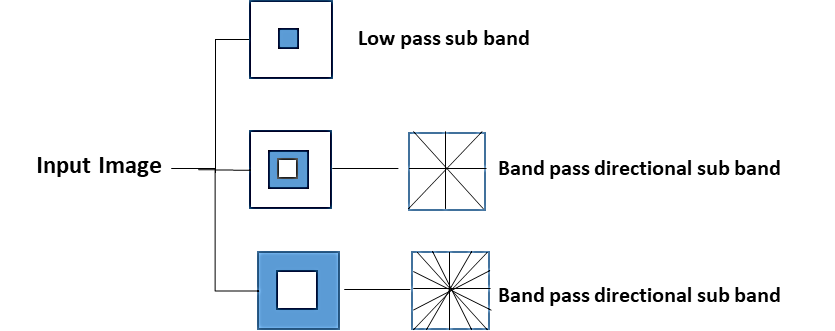 |
|  | Idealized frequency partitioning | 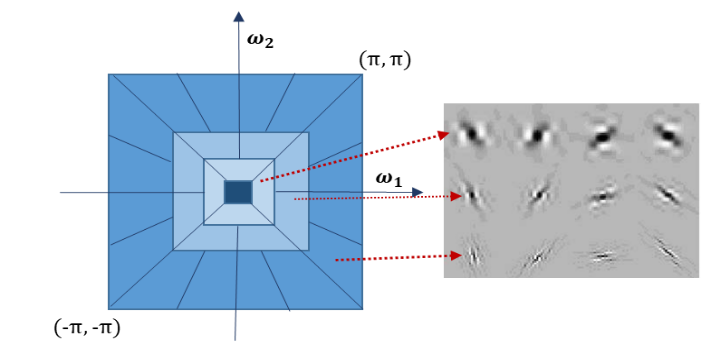 |
| Shearlet (NS) | Note | 1. Similar to Contourlet but instead of directional filters, shearlet filters are used. 2. Steps: (a) Multi-scale decomposition (achieved by NSP), (b) Localization of orientation (accomplished by a shearlet Filter) |
|  | Filter Bank | 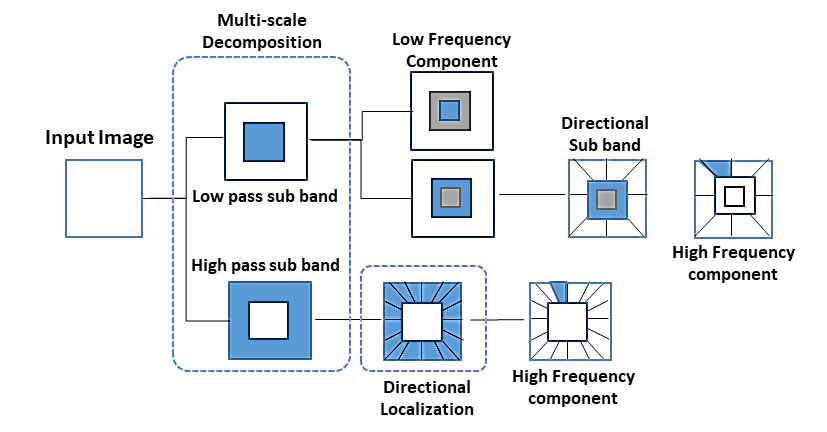 |
| Circlet | Note | 1. A robust tool to detect circular objects in images without the need for image segmentation 2. Decomposes an image to a set of circles with different radii using a Discrete Fourier Transform (DFT) filter bank 3. Describing the circlet parameters by a central position (x0, y0), radius (r0), and central frequency content (f0) 4. Obtaining all circlet components (Cμ(x, y)) by a shift or change in radius and central frequency in a reference circlet (Cref(x, y)) |
|  | Basis Functions | 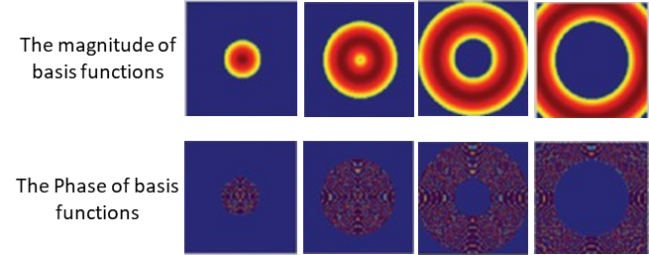 |
|  | Idealized frequency partitioning | 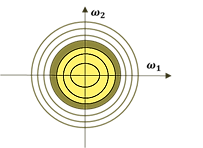 |
| Ellipselet | Note | 1. An extension of the circle transformation with elliptic basis functions 2. With four basis functions in four different directions at angels 0°, 90°, −45°, and 45° |
|  | Basis functions | 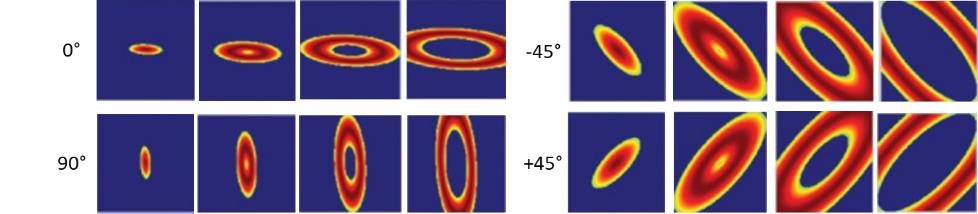 |
|  | Idealized frequency partitioning | 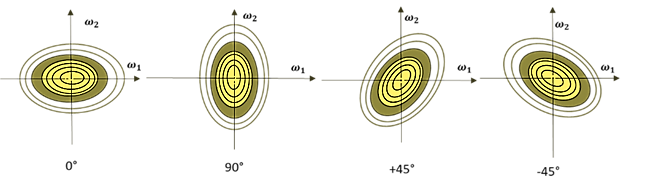 |


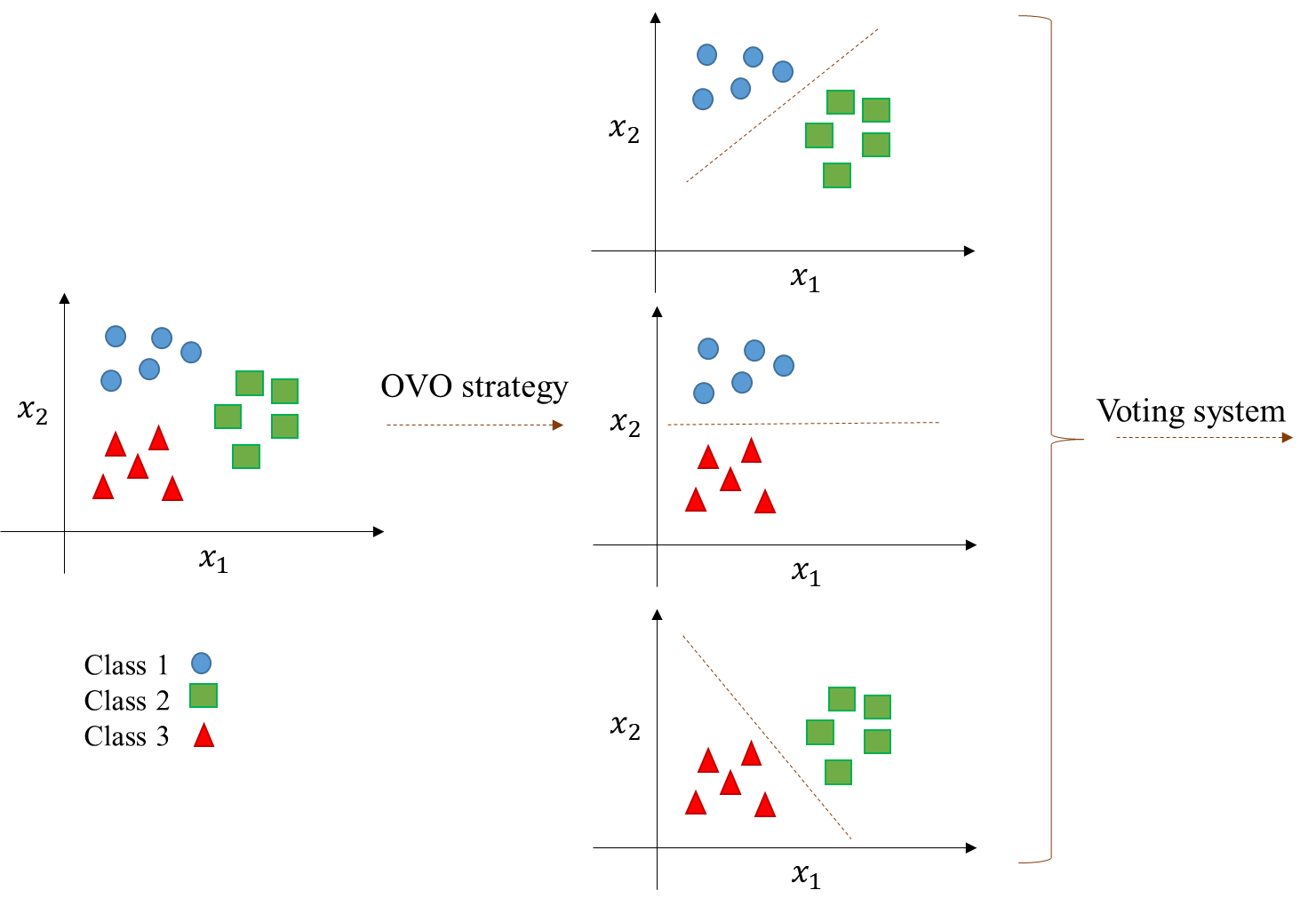


Figure-2. OVO strategy in MSVM

Table-3. Hyper-parameters selected for different kernels by Grid Search algorithm

| **kernel** | **Hyper-parameter** | **Tuned value** |
| --- | --- | --- |
| ^RBF^ | ^C^ | 100 |
|  | ^gamma^ | 10^-7^ |
| ^Linear^ | ^C^ | 0.01 |
| ^Polynomial^ | ^C^ | 10 |
|  | ^Degree^ | 2 |
| ^Sigmoid^ | ^C^ | 10 |
